# Defining when specialised mental health care is needed: a focus group study

**DOI:** 10.3399/bjgpopen20X101004

**Published:** 2020-01-08

**Authors:** Marit Nymoen, Eva Biringer, Jostein Helgeland, Harald Bjarne Hellesen, Liv Alsaker Sande, Miriam Hartveit

**Affiliations:** 1 Psychologist, Department of Research and Innovation, Helse Fonna Local Health Authority, Haugesund, Norway; 2 PhD Student, Department of Global Public Health and Primary Care, Faculty of Medicine, University of Bergen, Bergen, Norway; 3 Senior Researcher, Department of Research and Innovation, Helse Fonna Local Health Authority, Haugesund, Norway; 4 Local Medical Officer, Haugesund Health Care Office, Haugesund Municipality, Haugesund, Norway; 5 GP, Suldal General Practitioners Centre, Suldal, Norway; 6 Service User Representative, ADHD Norge (Service User Organization), FOUSAM, Haugesund, Norway; 7 Researcher, Department of Research and Innovation, Helse Fonna Local Health Authority, Haugesund, Norway; 8 Associate Professor, Department of Global Public Health and Primary Care, Faculty of Medicine, University of Bergen, Bergen, Norway

**Keywords:** continuity of patient care, general practice, mental health services

## Abstract

**Background:**

Shared understanding between GPs and hospital specialists concerning when patients need specialised mental health care is important to ensure patients receive appropriate care. The large amount of rejected referrals often indicates a lack of such shared understanding.

**Aim:**

To explore how patient representatives, GPs, and mental health specialists understand ‘need for specialised mental health care’, meaning that primary care is no longer sufficient.

**Design & setting:**

This qualitative study was conducted in western Norway. The study has a service user-involved research design in which GPs and patient representatives participated in all stages of the research process.

**Method:**

Six semi-structured focus group interviews were conducted. The groups were homogenous as they included only the perspectives of either GPs, mental health specialists, or patient representatives. Data were analysed using thematic analysis.

**Results:**

The need for specialised mental health care was assessed using two continuums: (a) the patient’s level of functioning and symptoms; and (b) characteristics of the healthcare system and the patient’s informal support networks. Assessment along these continuums were often overruled by the evaluation of expected usefulness of specialised mental health care. In addition, all participants reported they often adapted their definition of need to fit other stakeholders’ interpretations of need.

**Conclusion:**

Evaluation of need for specialised mental health care is complex and depends on several factors. This may explain some of the current challenges that exist with regard to equity and timely access to appropriate healthcare interventions.

## How this fits in

There is a paucity of studies looking at the views of GPs and other relevant stakeholders with regard to when secondary mental health care is needed. The study showed that the interplay of factors relevant for assessing need was complex and weighted somewhat differently among stakeholders. Existing guidelines for patient prioritisation may be inadequate for determining the best use of specialised mental health care. A fluid, customised definition of need may be necessary for optimal use of the limited resources available in specialised mental health care.

## Introduction

Patients suffering from mental illness often receive health care from several care providers.^1^ The responsibility for providing healthcare services is commonly transferred between healthcare levels.^2,3^ Processes where information, responsibility, and accountability for patients are assigned between healthcare providers is termed 'clinical handover'.^4^ Well-performed clinical handover is crucial for safe and reliable healthcare services.^4–6^ Transfer of sufficient information, shared understanding among healthcare professionals and a good working environment are important predictors of safe handover.^4–6^

An important and frequently occurring handover situation in many countries is the shift from primary care to specialised secondary care.^2,3,7,8^ Efforts have been made to improve the safety of patient handover between GPs and hospital specialists;^5,9,10^ however, evidence suggests that the main means of communication — referral letters — do not provide hospital specialists with the information that they need to prioritise patients for care.^2,8,11^ It has also been shown that factors such as the GP’s skills and interest may affect whether a patient receives help in primary or secondary mental health care.^12^ Greater use of specialised secondary health care and a consequent increase in clinical handover creates a growing risk of compromised patient safety in healthcare services.

Shared understanding concerning when secondary care should be offered is crucial, as patients receiving mental health care often need sequences of specialised care in addition to primary mental health care.^1^ Healthcare professionals often overestimate the level of shared understanding and knowledge concerning different processes in healthcare services.^13^

The two-tier healthcare system with primary and secondary level used in Norway, as many other countries, implies a major handover situation.^3,7,10^ The GPs are usually given a gatekeeper role between the two bodies of services and the decision of access to specialised mental health care is made by hospital specialists.^2,7,11^ In Norway, priority setting is regulated by legal acts and guidelines that emphasise the severity of the patient’s illness and the expected impact of the treatment on the patient’s quality of life.^14,15^ This results in a cost–benefit assessment.^14,15^ The guidelines recommend that priority should be given to patients who are, for example, caring for young children, aged <23 years, or currently on sick leave.^14^ Nevertheless, the criteria used to determine when patients need specialised mental health care are unclear.^11^

A Norwegian survey found that two-thirds of GPs and mental health specialists experience a lack of shared understanding regarding which patients should be accepted into specialised mental health care.^11^ Lack of agreement may lead to under- or over-use of these services, both of which are currently major challenges for the healthcare system^16^; however, both under- and over-use of mental healthcare services are poorly defined.^17^ In addition, the interpretation of ‘need’ should be influenced by the patients themselves, which is consistent with a recovery-oriented view of health care.^18^ Given the lack of shared understanding between stakeholders, it is essential to understand the criteria that are relevant for determining when a patient needs specialised mental health care as a result of primary health care no longer being sufficient. No previous studies exploring this issue were found by the present authors.

This study aims to investigate how representative groups of GPs, patients, and mental healthcare specialists perceived and defined need for specialised mental health care, and how the definitions corresponded among the groups. The authors aimed to explore what constitutes the point at which primary health care was no longer sufficient for the patient and specialised mental health care was deemed necessary.

## Method

This qualitative study was conducted within a critical theory paradigm.^19^ Focus group interviews were chosen as the method of data collection to provide rich information on the complexity of assessing need for specialised mental health care. This method enabled investigation of potential differences in stakeholders’ perceptions.^20,21^ The study was conducted and documented in accordance with the Standards for Reporting Qualitative Research (SRQR) and Consolidated Criteria for Reporting Qualitative Research (COREQ) guidelines.^22,23^

The research group involved in the present study comprised two GPs with extensive experience, one patient representative, two full-time researchers, and a researcher who was a PhD student and clinical psychologist. The latter three researchers were employed by the local health authority. One patient representative contributed in the planning stage, but could not participate further because of illness. In this service user-involved research design,^24,25^ all group members participated in all stages of the research process. This included developing the interview guide, recruiting participants, analysing the data, and writing the article. Three formal meetings were held, discussing the design of the study and analysing the results.

### Setting

This study was conducted in the region of the Helse Fonna Local Health Authority on the west coast of Norway. The catchment area includes approximately 180 000 citizens.^26^ In the 17 municipalities of this region, there are several types of community health and social services, such as out-of-hours medical services and mental health support teams. Three different centres provide specialised mental health care. It includes both in- and out-patient care, and ambulatory services.

### Sample

Focus group interviews were conducted with GPs, hospital specialists, and patient representatives. Characteristics of the focus groups are shown in Table 1.

**Table 1.** Focus group interview participants

Group number	Participants, *n*	Participant type
1	6	General practitioner
2	4	General practitioner
3	3	Hospital specialist in secondary care
4	3	Hospital specialist in tertiary and secondary care
5	3	Patient representative
6	4	Patient representative
**Total**	23	

All participants were recruited from the same region to enable discussions within the same context. Patient representatives and GPs were recruited using a snowball sampling method.^27^ One of the co-researchers proposed nine patient representatives from local mental health service user organisations. These nine representatives were then contacted individually and invited to participate in this study by email and telephone; two of the nine representatives declined to participate. All patient representatives had experience as a patient receiving mental health care or had a close relative receiving mental health care.

GPs were recruited from the local GP network. Hospital-based mental healthcare specialists (*n* = 16) were nominated by the leaders of relevant units, after which they were individually invited to participate. Ten of the 16 invited hospital specialists were not able to attend the focus group interview. The inclusion criterion for healthcare professionals was ≥1 year of experience as a GP or mental health specialist. Homogeneity of participant type in the focus groups was preferred. It was thought that this would facilitate participants’ full engagement in the discussion, as a mixed group setting may be affected by an unequal distribution of power between stakeholders.^21,28^

All participants were informed about the project and signed a written consent form before the focus group interviews. Participants were offered standard fees as defined by the Norwegian Medical Association (approximately 400 EUR)^29^ if they were interviewed outside of work hours. Three participants received this reimbursement.

### Data collection and analysis

In February–May 2018, six focus group interviews were conducted. The groups were homogenous as they included only groups of either GPs, hospital specialists, or patient representatives. Two interviews were held for participants from each of the stakeholder groups. The interviews were conducted in healthcare settings, mostly at local health authority facilities. The semi-structured interview guide included the following three main questions: (1) what clinical features of the patient or situational factors should be accepted by specialised mental health care?; (2) what characterises situations where you are unsure whether a patient should be accepted into specialised mental health care?; and (3) when are patients not in need of specialised mental health care? The interview guide was altered to suit each group of participants (for example, in the use of clinical terms or not) but the main questions remained the same.

During the focus group interviews, participants engaged in open dialogue where they could express what they saw as important in understanding the concept ‘need for specialised mental health care’. A moderator led all group interviews. Another researcher observed the interviews and took brief notes. Each interview lasted 60–90 minutes. Interviews were audio-recorded with participants’ consent and transcribed verbatim.

The results were analysed using thematic analysis.^30^ Each member of the research group familiarised themselves with the interview transcripts, and coded transcripts for meaningful content and themes before discussing the results in a consensus meeting.^30^ NVivo (version 12) was used for the transcript analysis. All authors provided a written description of their coding and thematic analysis. A meeting to finalise consensus regarding the results and writing the article was held in April 2019. Quotes were translated by professionals and evaluated by a native English-speaking research colleague.

## Results

The six focus group interviews, including GPs (*n* = 10), hospital specialists (*n* = 6), and patient representatives (*n* = 7; see Table 1) revealed four elements that were seen as relevant in the assessment of need for specialised mental health care, meaning that primary health care was no longer sufficient for the patient’s needs. The participant groups had different ways of understanding the themes, and described the themes using dissimilar concepts. Participants also expressed uncertainty concerning the roles and tasks of primary care and GPs in relation to hospital mental healthcare specialists. Box 1 presents a list of examples of what constituted need for specialised mental health care.

Box 1 **Examples of information in each theme from the three different perspectives**

**Table:** 

	Patient representatives	General practitioners	Hospital specialists
**The patient’s symptoms and functioning**	Patient is unable to function in daily life or unable to care for children. Substance abuse, trauma, psychosis, anxiety, depression, self-harm, suicidality, reckless or extreme behaviour, and eating disorders.	Worsening of the patient’s mental illness. Patient’s motivation and cognitive capacity for treatment. Needs a specialist’s help for evaluation of medicine and diagnosis. Treatment options only in specialised health care. Patient is unable to care for children or attend work. Patient lacks or has an unsustainable network.	Patient has deteriorating relationships. The patient’s symptoms (risk for suicide, psychosis). The patient’s daily functioning level.
**Contextual factors**	Patient has a fragile or lacking network.	Geographical factors and infrastructure. Patient has a fragile or lacking network. Patient has a tired family. Limitations of the GP’s competence and confidence.	Patient lacks housing. Patient’s upbringing. Patient’s network or family. If request is made during night or weekend. Patient living far away from SMHC, availability of transport. The GP cannot defuse the situation.
**The ‘adaptation process’**	GPs are not regarded as a part of mental health care. There is a lack of time at the GP’s office.	The GP has to adapt the definition of need to fit the specialist’s definition.	Professional medical discretion concerning the patient’s upbringing, personal history.
**Expected helpfulness**	Patient’s earlier experiences of treatment in SMHC. Patient’s actual or expected effect of treatment from SMHC.	Availability of treatment in primary mental health care or private clinics. Estimated time on waiting list for the patient.	Cost–benefit assessment. Patient history of treatment and effect of treatment in SMHC. Risk of the patient having no effect from treatment in SMHC.

SMHC = specialised mental health care.

Two themes were descriptions of the patient and their context. The first theme included specified patient characteristics, such as symptoms and level of functioning, that indicated specialised mental health care would be useful for the patient. The second theme comprised contextual characteristics, including the patient’s network and the services available for that patient. The last two themes represented prerequisites for evaluating the need for specialised care. The participants said that their definition of need was adjusted to increase the chances of achieving the desired outcome. The adjustment of the definition was described as an interactive process where patients and GPs were aware that hospital specialists had a different definition of need for specialised mental health care, and adjusted their communication to increase the chances of the patient receiving care. The fourth theme concerned the expected effect of treatment, as a key criterion for interpreting that there was a need for specialised care. Participants’ descriptions for each theme are presented below in more detail.

### The patient’s symptoms and functioning

Patient representatives most often described need for specialised mental health care in terms of cases where the patient was not able to function in daily life. They also mentioned typical signs of mental illness and diagnoses. The patient’s ability to take care of their family, to work, or to meet other obligations was also highlighted as a feature that determined whether the patient should receive specialised mental health care. One patient representative said:

' […] *you're not able to cope. For instance, a mother who must be a mother* […] *Family, work — many things that we just assume we can manage when we're healthy*.' (Patient representative)

In general, GPs noted changes in the patient’s functioning and symptoms of mental illness as key reasons for a patient needing specialised care. Participating GPs further emphasised that the patient’s motivation for treatment and likelihood of benefiting from the provided care were important patient characteristics to consider when deciding if a patient needed such care:

' […] *there is motivation and basically the intellectual capacity for receiving psychotherapy*.' (General practitioner)

In addition, GPs explained that a patient’s symptoms may call for a specific intervention that only specialised mental health care can offer, such as diagnostic evaluation or assessments requiring specialist competence. Hospital specialists emphasised that a risk of self-harm, risk of harming others, or symptoms of psychosis were some of the most important elements in the evaluation of need for specialised care; however, they mainly described need for specialised mental health care in the same way as legal regulations were written; that is, how need was described in legislation and priority guidelines concerning the right to specialised mental health care. One psychiatrist mentioned this early in the interview:

'*Strictly speaking, it is the first concern we must address. We have to consider whether the cost of implementing treatment is in contrast to or in proportion to the anticipated effect and anticipated benefit*.' (Hospital specialist)

### Contextual factors

Contextual factors included characteristics of the patient’s surroundings as well as healthcare system characteristics, including barriers to care in terms of geographical distance or physical access to mental healthcare services. All participant groups mentioned factors relating to the patient’s family and social network that they thought would affect that patient’s experience of need for specialised mental health care. GPs and patient representatives considered patients needed specialised mental health care if they had support networks that were fragile or lacking, or where relatives and networks were exhausted. One GP said:

' […] *if it is unsustainable … In other words, if the environment they* [the patient] *are a part of can no longer support them, and their network is disintegrating* […] *then I would make a referral*.' (General practitioner)

Most GPs mentioned that they wanted to treat their patients themselves, but would refer the patient to specialised mental health care if cooperation with the patient was difficult. They also reported that the level of confidence in treating patients with mental illnesses varied among GPs. Some hospital specialists noted that a patient’s existing network and support may be reasons for not prioritising that patient. Hospital specialists noted deteriorating relationships and a lack of housing as key reasons for admitting patients into specialised mental health care. These specialists also mentioned that, because of the small catchment area, knowledge of the patient’s upbringing and network mattered in their evaluation of need for such care.

Geography and infrastructure also played a key part in the evaluation of need. For example, GPs did not want to refer a patient if that meant long journeys to the hospital, often by public transport. This made it more difficult for GPs to ensure that patients received the help they needed. Hospital specialists reported that they accepted patients more readily into specialised care if the doctors at emergency services or the GP could not defuse the situation and when all possible solutions to the patient’s problem had already been tried in primary health care:

*'If the referral indicates, for instance, that there has been an attempt at closer follow-up by the general practitioner, or by municipal services, or if municipal services are already in place, and there is still no change, then we can attempt to treat them* [the patients].' (Hospital specialist)

### The ‘adaptation process’

The participants described an adaptation of the description of the patient’s needs in different ways to comply with other stakeholders’ assessment criteria, and by that increase their chances of a desirable outcome. GPs presumed that hospital specialists defined need for specialised mental health care differently to the patient and the GPs themselves, and therefore altered their definition when communicating with hospital specialists. Some GPs said that certain words or phrases increased the chances of admittance, such as implying that a person might have a serious condition:

'*Question of bipolar disorder.'* (General practitioner)

*'Young children, mental health problems*.' (General practitioner)

Participating hospital specialists indicated that they evaluated most referrals using professional medical discretion, and noted that there were no clear-cut ways to evaluate referrals. They also noted that the way in which referral letters were written was a key consideration in how they prioritised patients. For example, the referral letter should have sufficient information about the patient, such as information about their social network and ability to work. According to hospital specialists, a referral could be rejected because of a lack of information.

Some patient representatives perceived that there was a difference between being referred to specialist mental health care for the first time versus being re-referred. In that context, the referral was adapted to the hospital specialist’s interpretation of need. It was reportedly easier to be referred when the GP and healthcare personnel in specialised mental health care knew about the illness from a previous encounter with the patient. In addition, the way in which patients explained their symptoms to their GP reflected adaptation to the situation. In particular, patient representatives explained that they might adapt their description of symptoms and daily functioning in order to avoid interventions at a specialised healthcare level. One patient representative said that he sometimes omitted information about the reality of his day-to-day life when speaking with his GP:

*'* […] *you go to your general practitioner, you don't dare say how bad it really is, so you tend to downplay it*.' (Patient representative)

### Expected usefulness of specialised mental health care

Both GPs and patient representatives highlighted that there may be a need for more help than was currently received, but this was not necessarily a need for specialised mental health care. The experience of need for specialised mental health care depended on whether these services could provide help they expected to be available and useful. For example, one patient representative noted that receiving only pharmacological treatment was not seen as useful:

' […] *I feel as though a lot of treatment involves medication at these specialised mental healthcare centres. It seems that way*.' (Patient representative)

Based on GPs’ earlier experiences of insufficient responses to patients’ needs from specialist mental health care, solutions other than a referral to specialist care were noted as relevant options. They do not refer patients to specialised mental health care if they expect the existing primary healthcare services to provide care interventions that are equally or better suited to the patient’s situation and needs. Being referred to specialised mental health care may even harm patients:

'*You often stop to consider, as a general practitioner, whether you think a patient will be treated at all, or if they will be rejected* […] *because you risk disappointing a patient that may become even more ill because of the rejection. So then you have to re-evaluate this and discuss it with the patient*.' (General practitioner)

Estimated waiting time and the type of treatment available in specialised mental health care played key roles in deciding if there was need for such care. Most GPs said that the threshold for receiving specialised mental health care was too high. In their view, specialised services were not the optimal service provider for some patients because of long waiting lists and uncertainty concerning the prioritisation process. One GP stated that, in his opinion, the usefulness of treatment in specialised mental health care varied. Some hospital specialists were concerned that if a patient was admitted without benefiting from specialised care, the patients may experience it as a failure of care. In such cases, hospital specialists evaluated the patient’s potential rather than the usefulness of the treatment options in specialised mental health care. One hospital specialist said:

*'* […] *we also have people who are theoretically entitled to treatment* […] *they may perhaps have had numerous previous attempts at treatment with no effect, and this has changed nothing for them.* […] *this is a way for them to experience yet another potential failure*.' (Hospital specialist)

## Discussion

### Summary

This focus group study explored how ‘need for specialised mental health care’ was perceived by GPs, patient representatives, and hospital specialists, respectively. The results indicated agreement among stakeholders concerning four main themes, but stakeholders’ views varied within these themes. The patient’s symptoms and functioning, their network, and factors related to the overall healthcare system were important when evaluating if a person needed specialised mental health care. The interpretation of need for specialised mental health care was also dependent on the expected usefulness of treatment in specialised services and an ‘adaptation process’ among all three stakeholders.

### Strengths and limitations

To the authors' knowledge, this study was the first to explore the interpretation of need for specialised mental health care from the perspectives of different stakeholders. It provides new insight into how the 'border' between primary and secondary health services can be defined differently by patients and healthcare professionals. Focus group interviews can provide complex and rich data from each perspective.^20^ Methodologically, focus group interviews may not provide exhaustive data, but this method can be used to elicit new information concerning the research field in question.^20^ The inclusion of relevant stakeholder groups during different phases of the study strengthened the internal and external validity of the study, and reduced the chance of one perspective dominating the results. Reflexivity was emphasised and researchers aimed at weighting the perspectives of all participant groups equally in both the analysis and the writing of the article. It was aimed to recruit representative groups of participants; nonetheless, the use of snowball sampling^28^ might have affected the results in unpredicted ways, as there was no control over participant characteristics other than their reported previous experiences.

The main aim for the present study was to explore how criteria for being in need of a higher level of care is interpreted by stakeholders, by the example of handover between primary care and specialised mental health care in Norway. The organisation of health care differs between countries^7^; however, it is argued that the main findings are highly relevant for understanding handovers from one level to the next and also in other contexts.

A GP participated in developing the interview guide, the interviews, and the analysis. This could have introduced a conflict of interest, as that GP might have wished to emphasise certain views in both the interviews and analysis.

### Comparison with existing literature

The present results resonate with a survey that showed it is challenging for both GPs and hospital specialists to determine when patients should receive specialised mental health care.^11^ The findings were also consistent with earlier international studies that highlighted the need for better communication and cooperation among healthcare professionals.^31–34^ There may be an asymmetrical relationship between GPs and hospital specialists, which makes the handover process more complex.^31–34^ Previous studies also indicated there was a lack of shared understanding among hospital specialists regarding when specialised mental health care is needed.^3,9^

Contrary to the present results, an earlier report highlighted the quality of referral letters as a main reason for inefficient use of services^11^; however, in this study, the quality of referral letters was infrequently mentioned as a reason for rejecting patients. Uncertainty concerning when patients need specialised health care may explain some of the phenomena described in other research, such as variation in number of referrals to specialised mental health care.^12,35^

### Implications for research and practice

The results showed that it is challenging to identify when primary mental health care is insufficient for a patient and when there is a need for specialised mental health care. A clear definition of the service role may be beneficial for all stakeholders; however, given the range of factors that are relevant for determining the appropriate level of care for a patient, it is not believed that the comprehensive assessment by GPs can be fully standardised. Participants’ comments suggested that, currently, the evaluation of need was both comprehensive and dependent on the person’s situation. Existing guidelines for patient prioritisation, focusing mainly on the intensity and frequency of symptoms and level of functioning,^14,36^ may be inadequate for determining the best use of specialised mental health care. A fluid, customised definition of need may be necessary for optimal use of the limited resources available in specialised mental health care. A shared understanding is essential to increase predictability for all stakeholders and reduce the amount of resources used to handle referrals. The present study reveals a somewhat diverging view on the criteria for being in need of specialised mental health care. It may be advantageous to include the views of all three stakeholder perspectives to a larger degree than is presently the case when defining if such care is needed. The authors recommend incorporating the patient perspective as a mandatory part of the process where the mental health specialist considers the priority of the patient.

The results suggested that professional medical discretion played a significant role in the evaluation of need for specialised mental health care. The fact that professional medical discretion includes subjective evaluation may affect the horizontal equity of such services, as similar patients are evaluated differently. Participating GPs reported that they had to frame their referral letters in certain ways to increase a patient’s chances of receiving specialised care. This ‘adaptation process’ can skew the interpretation of need for specialised mental health care and lead to over- or under-use of these services. It may be necessary to re-evaluate the referral process and the communication means between GPs and specialised mental health care, in order to provide an equitable healthcare service. Further studies should aim to reveal how shared understanding between GPs and hospital specialists can be improved in the referral process to avoid under- or over-use of specialised health care.

## Trial registration

Norwegian Centre for Research Data (reference number: 56158).
